# Gastrointestinal Assessment and Therapeutic Intervention for the Management of Exercise-Associated Gastrointestinal Symptoms: A Case Series Translational and Professional Practice Approach

**DOI:** 10.3389/fphys.2021.719142

**Published:** 2021-09-07

**Authors:** Stephanie K. Gaskell, Christopher E. Rauch, Ricardo J. S. Costa

**Affiliations:** Department of Nutrition, Dietetics and Food, Monash University, Notting Hill, VIC, Australia

**Keywords:** circulatory-gastrointestinal, neuroendocrine-gastrointestinal, orocecal transit time, gastrointestinal symptoms, gastric motility, gastroparesis, ileus, intestinal epithelium

## Abstract

This translational research case series describes the implementation of a gastrointestinal assessment protocol during exercise (GastroAxEx) to inform individualised therapeutic intervention of endurance athletes affected by exercise-induced gastrointestinal syndrome (EIGS) and associated gastrointestinal symptoms (GIS). A four-phase approach was applied. Phase 1: Clinical assessment and exploring background history of exercise-associated gastrointestinal symptoms. Phase 2: Individual tailored GastroAxEx laboratory simulation designed to mirror exercise stress, highlighted in phase 1, that promotes EIGS and GIS during exercise. Phase 3: Individually programmed therapeutic intervention, based on the outcomes of Phase 2. Phase 4: Monitoring and readjustment of intervention based on outcomes from field testing under training and race conditions. Nine endurance athletes presenting with EIGS, and two control athletes not presenting with EIGS, completed Phase 2. Two athletes experienced significant thermoregulatory strain (peak core temperature attained > 40°C) during the GastroAxEx. Plasma cortisol increased substantially pre- to post-exercise in *n* = 6/7 (Δ > 500 nmol/L). Plasma I-FABP concentration increased substantially pre- to post-exercise in *n* = 2/8 (Δ > 1,000 pg/ml). No substantial change was observed in pre- to post-exercise for systemic endotoxin and inflammatory profiles in all athletes. Breath H_2_ responses showed that orocecal transit time (OCTT) was delayed in *n* = 5/9 (90–150 min post-exercise) athletes, with the remaining athletes (*n* = 4/9) showing no H_2_ turning point by 180 min post-exercise. Severe GIS during exercise was experienced in *n* = 5/9 athletes, of which *n* = 2/9 had to dramatically reduce work output or cease exercise. Based on each athlete’s identified proposed causal factors of EIGS and GIS during exercise (i.e., *n* = 9/9 neuroendocrine-gastrointestinal pathway of EIGS), an individualised gastrointestinal therapeutic intervention was programmed and advised, adjusted from a standard EIGS prevention and management template that included established strategies with evidence of attenuating EIGS primary causal pathways, exacerbation factors, and GIS during exercise. All participants reported qualitative data on their progress, which included their previously presenting GIS during exercise, such as nausea and vomiting, either being eliminated or diminished resulting in work output improving (i.e., completing competition and/or not slowing down during training or competition as a result of GIS during exercise). These outcomes suggest GIS during exercise in endurance athletes are predominantly related to gastrointestinal functional and feeding tolerance issues, and not necessarily gastrointestinal integrity and/or systemic issues. GastroAxEx allows for informed identification of potential causal pathway(s) and exacerbation factor(s) of EIGS and GIS during exercise at an individual level, providing a valuable informed individualised therapeutic intervention approach.

## Introduction

Exercise-associated gastrointestinal symptoms are a common feature of endurance exercise, and can vary in severity from a minor level of discomfort and inconvenience to severe symptoms of clinical significance. Consequently, GIS during exercise, may have either minimal or substantial negative impact on exercise performance, resulting in reduced workload, cessation of exercise and/or withdrawal from activity (i.e., recreational fitness, training, or competition) (Costa et al., 2017b, 2020b). The type of GIS during exercise experienced by endurance athletes frequently reported include: upper (i.e., gastro-oesophageal: upper abdominal bloating and/or pain, belching, gastric acidosis, urge to regurgitate, mild regurgitation to projectile vomiting), lower [i.e., flatulence, lower abdominal bloating and pain, urge to defecate, defecation with or without abnormalities (e.g., diarrhoea and/or faecal blood loss)], and other related symptoms (i.e., nausea, and acute transient abdominal pain) (Gaskell et al., 2019).

It is now well established that the underlying pathophysiology of GIS during exercise is multifaceted, and appears to stem from “*exercise-induced gastrointestinal syndrome*” (EIGS), involving two primary causal pathway models (i.e., circulatory-gastrointestinal and neuroendocrine-gastrointestinal pathways), and a potential third causal factor in form of mechanical strain (i.e., mechanical strain on the splanchnic arena through jarring, jolting, acute impact, friction, and/or body position). A comprehensive overview on EIGS, exacerbation factors, health and performance implication can be viewed in Costa et al. (2017b, 2020b). Clinical complications of EIGS can be acute, such as reversible colitis (Grames and Berry-Cabán, 2012; Benmassaoud et al., 2014), and/or gastrointestinal paresis (Gaskell et al., 2021b); or although uncommon, can even lead to fatal outcomes through luminal to circulation microbial and/or endotoxin translocation inducing septic shock (Gill et al., 2015a, b; Lim, 2018). The neuroendocrine-gastrointestinal pathway of EIGS appears to be the predominant causal pathway of acute GIS during exercise, compared with the circulatory-gastrointestinal pathway, which does not appear to play a consistent key role in evoking GIS during exercise (Costa et al., 2017a; Snipe et al., 2018a,b; Gaskell et al., 2021b). This pathway has been implicated in promoting gastroparesis with or without paralytic ileus during exercise, which appears to promote acute onset of severe GIS during exercise (Costa et al., 2019; Gaskell et al., 2021b). Anecdotally, ultra-endurance athletes (i.e., runners and/or cyclists) commonly complain of rapid onset of GIS during exercise ∼4 h into exercise, in which, there is now accruing laboratory research presenting disturbance to gastrointestinal function (e.g., motility, digestion and/or absorption) and feeding intolerance being the main instigators of this later onset GIS during exercise (Costa et al., 2017a, 2019; Alcock et al., 2018; Miall et al., 2018; Gaskell et al., 2020, 2021b; Russo et al., 2021a,b,c).

It is now well established that a minimum magnitude of exercise stress is required in order to perturb gastrointestinal integrity and function, invoke a systemic inflammatory and immune response including GIS during exercise to a level of clinical significance (i.e., values consistent with gastrointestinal inflammatory or functional diseases/disorders) or be performance limiting. It has previously been reported that the minimum threshold of exercise stress equates to ≥2 h at 60% V̇O_2__max_ in ≥35.0°C ambient temperature or ≥3 h at 60% V̇O_2__max_ in temperate conditions in laboratory controlled studies (Costa et al., 2017a, 2019, 2020a; Alcock et al., 2018; Miall et al., 2018; Snipe et al., 2018a,b; Gaskell et al., 2019, 2020, 2021b), and ultra-endurance competition in field studies (Bosenberg et al., 1988; Brock-Utne et al., 1988; Jeukendrup et al., 2000; Gill et al., 2015a, b). Anything less than this appears insufficient in provoking gastrointestinal disturbance that present clinical or performance implications and of little relevant to “real-world” application (Costa et al., 2017b, 2020b). Additionally, key extrinsic [i.e., modality, altitude, thermoregulatory modifiers, circadian variation, and pharmaceutical administration (e.g., NSAIDs)] and intrinsic (i.e., biological sex, hydration status, dietary intake, feeding tolerance, predisposition, and gut microbiota composition) factors appear to also play an important role in exacerbating GIS incidence, type and severity during exercise (Costa et al., 2017b, 2020b). In order to assess and measure the significance of exercise-associated perturbations to gastrointestinal integrity, function, and systemic responses linked to the primary causal pathways and secondary outcomes of EIGS, there are a range of assessment and analysis techniques that have been investigated. These may include, plasma cortisol for assessment of overall exertional stress, gastric tonometry for assessing perfusion associated ischaemia, plasma I-FABP for magnitude of enterocyte injury, dual-sugars test for intestinal epithelial permeability, systemic sCD14 and/or LBP for indirect markers of luminal bacterial endotoxin translocation, and specified plasma cytokine concentrations for systemic inflammatory profile (Gaskell et al., 2021a). In addition, lactulose challenge for orocecal transit time (OCTT), electrogastrography for paresis determination, non-metabolisable sugars challenge for intestinal epithelial transporter activity, and/or specific food challenges and breath H_2_ detection to establish carbohydrate malabsorption.

Considering that GIS during exercise is multifactorial in nature (Costa et al., 2016) it is clear that there is no “*one-size-fits-all*” approach in preventing and managing these gastrointestinal issues. Therefore, individualised assessment of GIS during exercise is imperative in order to determine the most effective prevention and management strategy specific to each individual athlete. There is substantial research into prevention and management strategies for EIGS and associated GIS, with such strategies showing varying ranges of effectiveness from being favourable, neutral or negative. Such prevention and management strategies include maintaining euhydration status (Costa et al., 2019), carbohydrate feeding during exercise (Snipe et al., 2017), gut training (Costa et al., 2017a), a diversity of carbohydrate recipe mixtures (i.e., multi-transportable carbohydrates, carbohydrate-protein co-ingestion, carbohydrate texture) (Jentjens et al., 2004; Rowlands and Clarke, 2011; Rowlands and Wadsworth, 2012; Haakonssen et al., 2014; Baur et al., 2016; Guillochon and Rowlands, 2017; Rowlands and Houltham, 2017), thermoregulatory strategies such as cooling (e.g., internal and external, pre and per) and heat acclimatisation/acclimation (Barberio et al., 2015; Lee et al., 2018; Snipe and Costa, 2018a), dietary fermentable oligo- di- mono- saccharide and polyols (FODMAP) modification (Gaskell et al., 2020), and acute or long-term nutritional supplementation [i.e., probiotic, specific amino acids (e.g., glutamine, arginine, citrulline, glycine, and tyrosine), bovine colostrum, anti-oxidants, curcumin, nitrate, etc.] (Costa et al., 2020b). There are wide discrepancies between study outcomes with these strategies due to differences in research methodology, such as, varying degrees of exercise stress models, experimental design, variability in supplementation interventions, confounder control, sample collection and analysis, limited and inconsistent scope of EIGS markers utilised, and heterogeneous populations. Due to research showing large individual variation in responses, broad-spectrum prevention and management strategies may not work for all athletes suffering from GIS during exercise as a result of EIGS. To date, no study has comprehensively assessed individual gastrointestinal responses to exertional or exertional-heat stress, and implemented an individualised action plan in the prevention and management of GIS during exercise, stemming from EIGS.

Establishing the causal and exacerbation factor(s) for a particular GIS during exercise is difficult without an individual tailored exercise gastrointestinal assessment due to the multifaceted and multilayers of EIGS (e.g., circulatory-gastrointestinal pathway, neuroendocrine-gastrointestinal pathway, mechanical instigators, extrinsic and intrinsic exacerbation factors). Therefore, the aims of this translational research case series were to: (1) Clinically assess athletes presenting severe GIS during exercise using retrospective exploration; (2) provide a prospective gastrointestinal assessment protocol during exercise (GastroAxEx), using previously established valid and reliable gastrointestinal assessment measurement tools that were used to inform an individualised therapeutic intervention for EIGS and GIS during exercise; (3) implement individualised therapeutic management interventions; and (4) assess outcomes of therapeutic management plans in training and competition, and adjust accordingly.

## Materials and Methods

### Presentation of Athletes

After ethics approval from Monash University Human Research Ethics Committee (24726), 12 athletes presented to the BASE Facility, Nutrition and Exercise Clinic, at Monash University (Melbourne, Australia) with GIS during exercise experienced during competition. However, nine recreational to elite level non-heat acclimatised endurance athletes met the inclusion criteria, provided informed consent, and volunteered to participate in the translational research. Each of the athletes were identified as experiencing EIGS and subsequent GIS during exercise (Table 1). The most common reported GIS was nausea and vomiting (*n* = 8/9) that negatively influenced feeding tolerance during exercise (i.e., unable to tolerate feeding and/or drinking during exercise), and in all cases (*n* = 9/9) resulted in reduced event workload and in the majority of cases withdrawal from competition (*n* = 5/9). Within the cohort, rapid onset of GIS during exercise commonly occurred later in exercise (≥3 h), with only two athletes (runners) occasionally experiencing GIS during exercise early onset during an event (1–2 h). Athletes participating in long course triathlon participation (*n* = 3), reported that GIS during exercise escalated on the run leg (*n* = 1 at 5–7 h and *n* = 2 at 9–12 h of total exercise duration), with milder GIS during exercise experienced on the bike leg. In addition, two amateur runners who typically experience minimal GIS during exercise and who undertook the GastroAxEx as part of the Monash University- BASE Facility, Nutrition & Exercise Clinic professional practice and research activities were included as control cases and for comparative purposes.

**TABLE 1:**
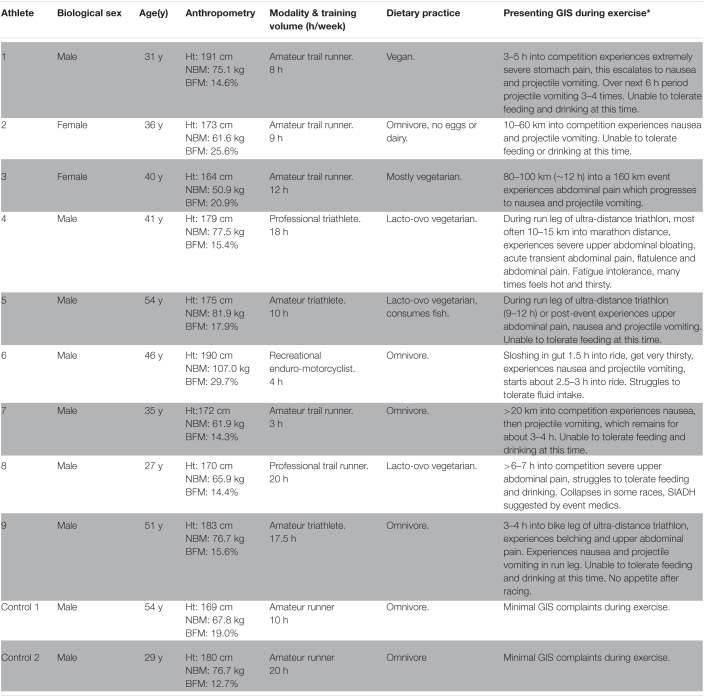
Case series and control participant characteristics and presentation of exercise-associated gastrointestinal symptoms (GIS).

BFM, Body fat mass; GIS, gastrointestinal symptoms; Ht, height; NBM, nude body mass; and SIADH, syndrome of inappropriate anti-diuretic hormone secretion. *Extreme GIS during exercise as determined by the exercise specific modified visual analogue scale (mVAS) (Gaskell et al., 2019). Control 1 and Control 2: runners with minimal GIS complaints during exercise.

### EIGS and GIS During Exercise Assessment and Management Procedures

**FIGURE 1:**
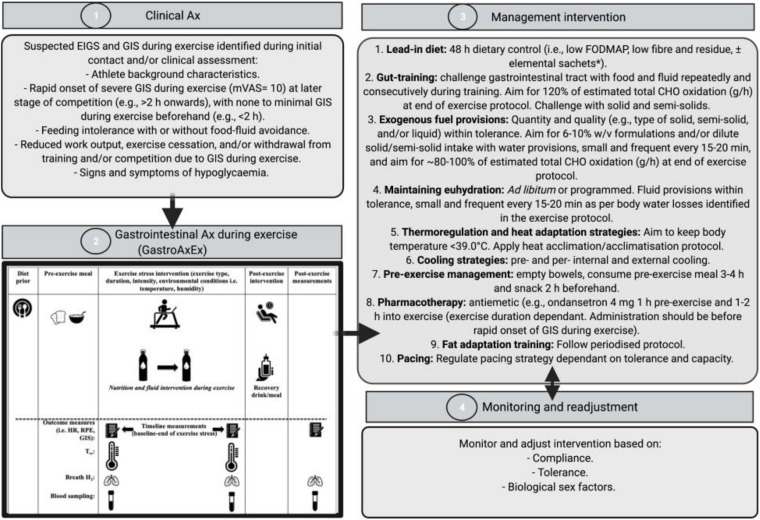
Four-phase EIGS and GIS during exercise assessment and management procedures. Elemental sachets: carbohydrate (CHO) = 60 g, protein (Pro) = 20 g, Energy = 325 kcal, Fat = 0 g, Fibre < 1 g, FODMAP: < 1 g. Ax, assessment; GastroAxEx, gastrointestinal assessment during exercise; GIS, gastrointestinal symptoms; HR, heart rate; RPE, rating of perceived exertion; TCR, thermal comfort rating; T_re_, rectal temperature. Intervention based on original research providing evidence of positive outcomes, and avoiding those showing evidence of neutral to negative outcomes, on markers of EIGS (e.g., functional, integrity, and systemic markers) and GIS during exercise (Costa et al., 2017b, 2020b). Adapted from Gaskell et al. (2021a), with permission.

EIGS and GIS during exercise assessment and management procedures have previously been described in Gaskell et al. (2021a), and are depicted in Figure 1 and consist of 4 distinct phases:

Phase 1: Clinical assessment of the athlete including specific details about their GIS during exercise (i.e., type, severity, onset, timing, modality, environmental conditions, pre- and during- exercise feeding and drinking habits). Suspected EIGS was identified if athletes met the following criteria: (1) rapid onset of nausea, urge to regurgitate, regurgitation at later stage of competition (e.g., >2 h onward), with no GIS during exercise beforehand (e.g., <2 h); (2) feeding and drinking intolerance with or without food-fluid avoidance; (3) reduced work output, exercise cessation, and/or event withdrawal due to GIS during exercise; and/or (4) signs and/or symptoms of hypoglycaemia (e.g., reduced gastrointestinal to systemic nutrient bioavailability).

Phase 2: A laboratory-controlled simulated gastrointestinal assessment during exercise (GastroAxEx), tailored to the individual, was designed and conducted (Table 2). The modality, exercise intensity and duration, environmental temperature, routine race/event nutrition preparation (i.e., 24–48 h prior to event), and during race/event nutrition was considered when designing the athlete specific laboratory-controlled simulation. Based on previously published research, the magnitude of exertional stress applied was in accordance with what typically provokes exercise-associated gastrointestinal disturbance of relative performance and clinical significance (Costa et al., 2017b, 2020b). Based on individual clinical assessment from Phase 1 appropriate gastrointestinal and physiological assessment markers were established and determined.

**TABLE 2:**
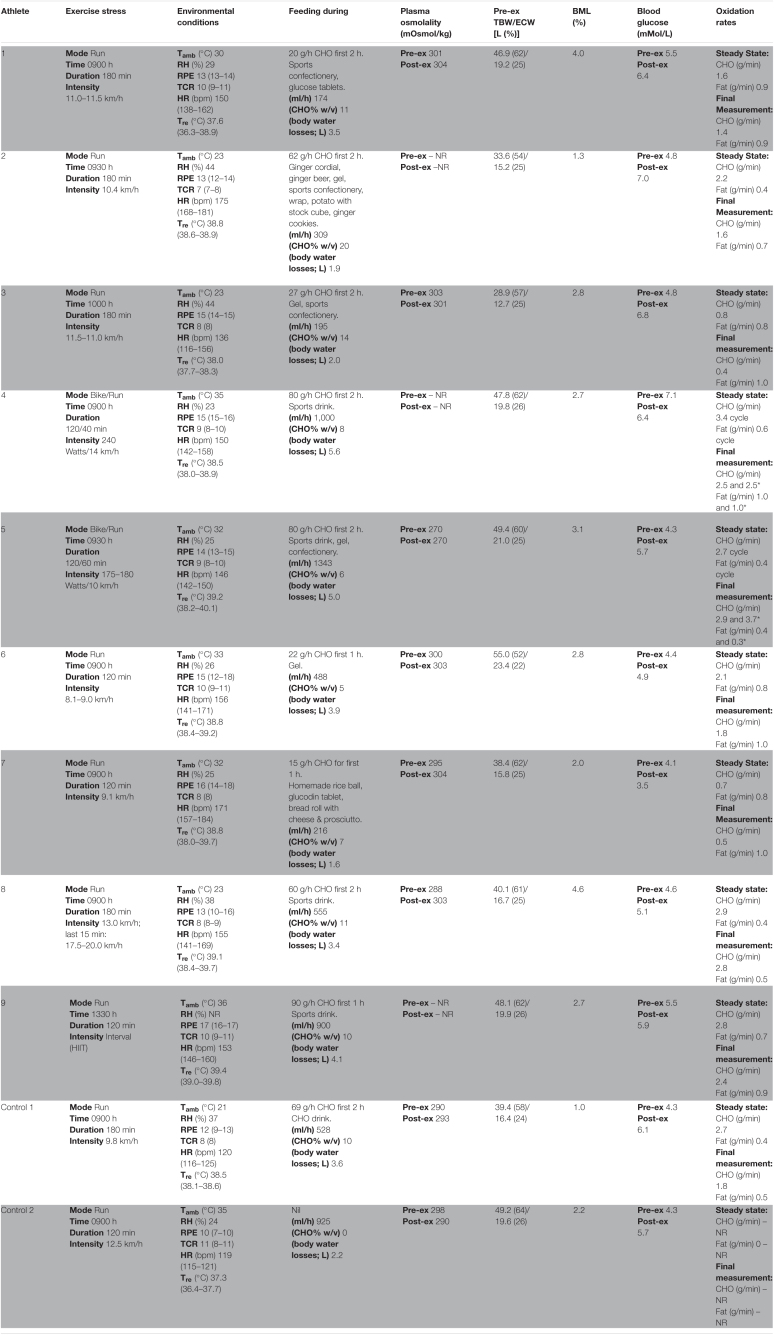
Individualised laboratory-controlled simulated gastrointestinal assessment during exercise (GastroAxEx) in endurance athletes experiencing EIGS with severe GIS and endurance athletes not experiencing EIGS and presenting minimal GIS (controls).

Mean (range, where applicable), otherwise specified. BML, body mass loss; CHO, carbohydrate; ECW, extracellular water; ex, exercise; HIIT, high intensity interval training (intervals, 2 min at 12.5 km/h; 3 min at 9.5 km/h; steady state running for 20 min at 10.5 km/h); HR, heart rate; T_amb_, ambient temperature; RH, relative humidity; RPE, rating of perceived exertion; TCR, thermal comfort rating; T_re_, rectal temperature; w/v, water volume equivalent; TBW, total body water. *Cycling and running.

Phase 3: Based on the outcomes of the GastroAxEx from Phase 2, an individualised therapeutic intervention phase was developed consisting of the following aims: (1) reduce pre-exercise food and fluid gastrointestinal load and/or burden; (2) maintain gastrointestinal patency during exertional stress; and (3) ameliorate physiological strain to exercise stress (e.g., thermal strain and euhydration maintenance). All intervention procedures (Figure 1) were practised in training and/or less important event/s, prior to using in targetted event/s. Intervention may have included the following: 48-h dietary control [i.e., low fermentable oligo- di- mono- saccharide and polyols (FODMAP), fibre and residue intake] ± elemental sachets (Gaskell et al., 2020), gut-training (Costa et al., 2017a; Miall et al., 2018), exogenous fuel provision within tolerance (Costa et al., 2017a; Snipe et al., 2017; Miall et al., 2018), hydration provision within tolerance (Costa et al., 2019), thermoregulation targetted and heat acclimation/acclimatisation strategies (Costa et al., 2014a), pre- and/or per- internal and/or external cooling strategies (Snipe et al., 2018a; Costa et al., 2020b), pre-exercise dietary management (Gaskell et al., 2020), pharmacotherapy application (i.e., ondansetron with medical collaboration and prescription, and NSAID avoidance) (Warden, 2010; Pasternak et al., 2018), fat adapt training and pacing strategies (Rauch et al., 2018), and also taking into account the biological sex of the athlete (Snipe and Costa, 2018b).

Phase 4: Monitoring and readjustment of intervention, which included checking the athlete was healthy leading into intervention, followed the dietary intervention, menstrual status where applicable (i.e., the female athlete), gut-training compliance, food and fluid intake during exercise tolerance, and thermal strain targetted strategy tolerance and compliance. In addition, measuring and recording GIS during exercise, physiological variables, and environmental conditions in training and/or competition where possible, checking other possible triggers (e.g., nutritional supplementation and anti-inflammatory medication), and assess whether further gastrointestinal assessment at rest and during exercise is warranted (i.e., medical procedures for gastrointestinal disease/disorder diagnosis, gut microbiota composition, luminal pathogenic assessment (e.g., fungal, bacterial, parasitic, and/or allergen), specific food allergen or intolerance) (Bate et al., 2010; Costa et al., 2017b; Bennett et al., 2020).

### Gastrointestinal Assessment During Exercise (GastroAxEx)

Figure 1 (part 2) and Table 2 depicts the laboratory controlled GastroAxEx protocol developed for each individual athlete based on their clinical presentation of EIGS and GIS during exercise. A running protocol on a motorised treadmill (Forma Run 500; Technogym, Seattle, WA, United States) was undertaken by seven athletes (i.e., trail runners and enduro-motorcyclist), and a cycle (i.e., participant’s own bicycle attached to a Wahoo KICKR cycle ergometer (Wahoo Fitness, Atlanta, GA, United States), previously validated in the power output range of all participants’ maximum aerobic power (MAP) (Zadow et al., 2016) + running simulation by two athletes (i.e., long course triathletes). All exercise protocols were at least 2 h duration with the longest 3 h 40 min. Six athletes undertook exercise in the heat (ambient temperature 30–35°C) and three in thermoneutral conditions (ambient temperature 23°C), as indicated by Phase 1.

### Sample and Data Collection – GastroAxEx

Athletes were asked to replicate their typical event preparation (i.e., dietary, exercise, and sleep habits) leading (1–2 days) into the GastroAxEx, this included an exercise taper (no strenuous exercise 48 h before testing) and their nutritional intake leading into (i.e., 24–48 h prior) and during competition (i.e., race nutrition). Athletes reported to the laboratory 1 h before exercise commencement, after consuming their typical pre-event meal or snack 2 h beforehand. A dietary log (1–3 days) determined their nutritional intake, as per previously established dietary intake assessment and analysis procedures (Costa et al., 2013a, b, 2014b). Participants were asked to void before nude body mass measurement, provide a breath sample into a 250 mL breath collection bag (Wagner Analysen Technick, Bremen, Germany), and complete a mVAS GIS assessment tool (Gaskell et al., 2019), as a baseline measure. Blood samples were then collected where indicated by venipuncture from an antecubital vein into lithium heparin (6 mL, 1.5 IU⋅ml^–1^ heparin) and K_3_EDTA (4 ml, 1.6 mg⋅ml^–1^ EDTA) vacutainers. Rectal temperature (T_re_) was monitored during exercise with participants inserting a thermocouple 12–15 cm beyond the external anal sphincter (Alpha Technics Precision Temperature 4600 Thermometer, Oceanside, CA, United States). Athletes then completed their individual tailored GastroAxEx (Table 2). Participants were provided with and instructed to consume their typical during competition food and fluid intake (i.e., race nutrition) along the exercise protocol, and were asked to refrain from this in the last 1 h of exercise testing due to orocecal transit time (OCTT) testing procedures. During exercise food and fluid intake was recorded in real-time. Water was available *ad libitum* throughout exercise. To determine OCTT, participants were provided with a solution containing 20 g of lactulose (Actilax, alphapharm, Qld, Australia), in the final 30 min of exercise. Breath samples were then collected immediately post-exercise and every 15 min during the recovery period, ranging from a 2 to 3 h timeline. The time interval between ingestion of lactulose and rise in breath hydrogen (H_2_) 10 ppm, with two consecutive readings above baseline (BL) was used as a measure of OCTT (Bate et al., 2010). The OCTT response was classified into the following categories: normal: >10 ppm (vs BL) at 30–60 min post-exercise (1:00–1:30 h post lactulose ingestion); slow: >10 ppm (vs BL) at 60–90 min post-exercise (1:30–2:00 h post lactulose ingestion); very slow: >10 ppm (vs BL) at 90–120 min post-exercise (2:00–2:30 h post lactulose ingestion), and absent response (indicative of suspected exercise-associated gastroparesis and/or paralytic (sub-paralytic) ileus): no or <10 ppm (vs BL) throughout 2–3 h recovery period (Gaskell et al., 2021b). T_re_, heart rate (HR), rating of perceived exertion (RPE), and thermal comfort rating (TCR) were measured every 30 min during exercise. Body mass, GIS, and feeding tolerance were measured every 30 min during exercise. Breath-by-breath indirect calorimetry (Vmax Encore Metabolic Cart, CaseFusion-BD, Franklin Lakes, NJ, United States) was used to measure *V̇*O_2_, *V̇*CO_2_, and RER for 5 min continuously every 20 min during exercise. Total non-protein carbohydrate and fat oxidation was determined from the equations of Péronnet and Massicotte (1991):

$$\mathrm{Total}\mathrm{carbohydrate}\mathrm{oxidation}:\left(4.585\text{×}\dot{V}{\text{CO}}_{2}\right)-\left(3.226\times \dot{V}{\text{O}}_{2}\right)$$

$$\mathrm{Total}\mathrm{fat}\mathrm{oxidation}:\left(1.695\times \dot{V}{\text{O}}_{2}\right)-\left(1.701\text{×}\dot{V}{\text{CO}}_{2}\right)$$

Immediately after exercise, a blood sample was collected where indicated. Nude body mass and GIS were recorded immediately post-exercise. Participants remained seated during the recovery period and consumed water *ad libitum*. GIS were recorded every 30 min during the 2–3 h post-exercise period. Participants were provided with a standard meal 2 h post-exercise in accordance with ethical procedures, as previously described (Russo et al., 2021a,b,c). Total body water, including extracellular water, was determined through an 8-point multifrequency bioelectrical impedance analyser (mBCA 515, Seca, Ecomed, Hamburg, Germany) before exercise and during the recovery period, where indicated.

### Sample Analysis – GastroAxEx

Breath samples (20 ml) were analysed in duplicate (CV: 3.0%) for hydrogen (H_2_) content using a gas-sensitive analyser (Breathtracker Digital Microlyzer, Quintron, Milwaukee, Wisconsin, United States). Whole blood haemoglobin was determined by a HemoCue system (Hb201; HemoCue, Ängelholm, Sweden), and haematocrit was determined by the capillary method with a microhematocrit reader (ThermoFisher Scientific), both from heparin whole blood samples. Haemoglobin and haematocrit values were used to estimate changes in plasma volume (P_V_) relative to baseline and used to correct plasma variables. Blood glucose concentration was measured pre, every 30 min during and post-exercise (Accu-Chek *Proforma*; Roche Diagnostics, Indianapolis, Ind., United States) (CV: 3.0%). The remaining heparin and K_3_EDTA whole blood samples were centrifuged at 4,000 rpm for 10 min within 15 min of sample collection. Plasma was aliquoted into 1.5 ml micro-storage tubes and frozen at −80°C until analysis, except for 2 × 50 μl heparin plasma that was used to determine plasma osmolality (P_Osmol_) in duplicate (CV: 0.5%) by freezepoint osmometry (Osmomat 030, Gonotec, Berlin, Germany). Plasma concentration of cortisol (DKO001, IBL International, Kiel, Germany), intestinal fatty acid binding protein (I-FABP) (HK406-02, Hycult Biotech, Uden, Netherlands), soluble CD14 (sCD14) (HK320-02, Hycult Biotech, Uden, Netherlands), LBP (HK315, Hycult Biotech, Uden, Netherlands) and claudin-3 (SEF293Hu, Cloud-Clone Corp., Katy, Texas, United States), were determined by ELISA. Plasma concentrations of IL-1β, TNF-α, IL-6, IL-8, IL-10, and IL-1ra were determined by multiplex ELISA (HCYTOMAG-60K, EMD Millipore, Darmstadt, Germany). All variables were analysed in duplicate as per manufacturer’s instructions, with standards and controls on each plate, and each participant assayed on the same plate. The CV for all ELISA markers were <10.4% and multiplex cytokine profile 8.1%.

### Data Presentation and Analysis

From a translational professional practice perspective, data are presented as full values for individual responses for all measured primary and secondary variables. In cases where primary or secondary variables were not collected, the *n* has been reported accordingly (e.g., *n* = 9/9). Individual participant raw values were compared against research established baseline and magnitude of exercise response reference ranges previously established indicative of performance and/or clinical (i.e., comparable to values reported in gastrointestinal tract functional and integrity diseases/disorders) significance or insignificance (Costa et al., 2017b, 2020b).

## Results

### Gastrointestinal Assessment During Exercise (GastroAxEx)

**FIGURE 2:**
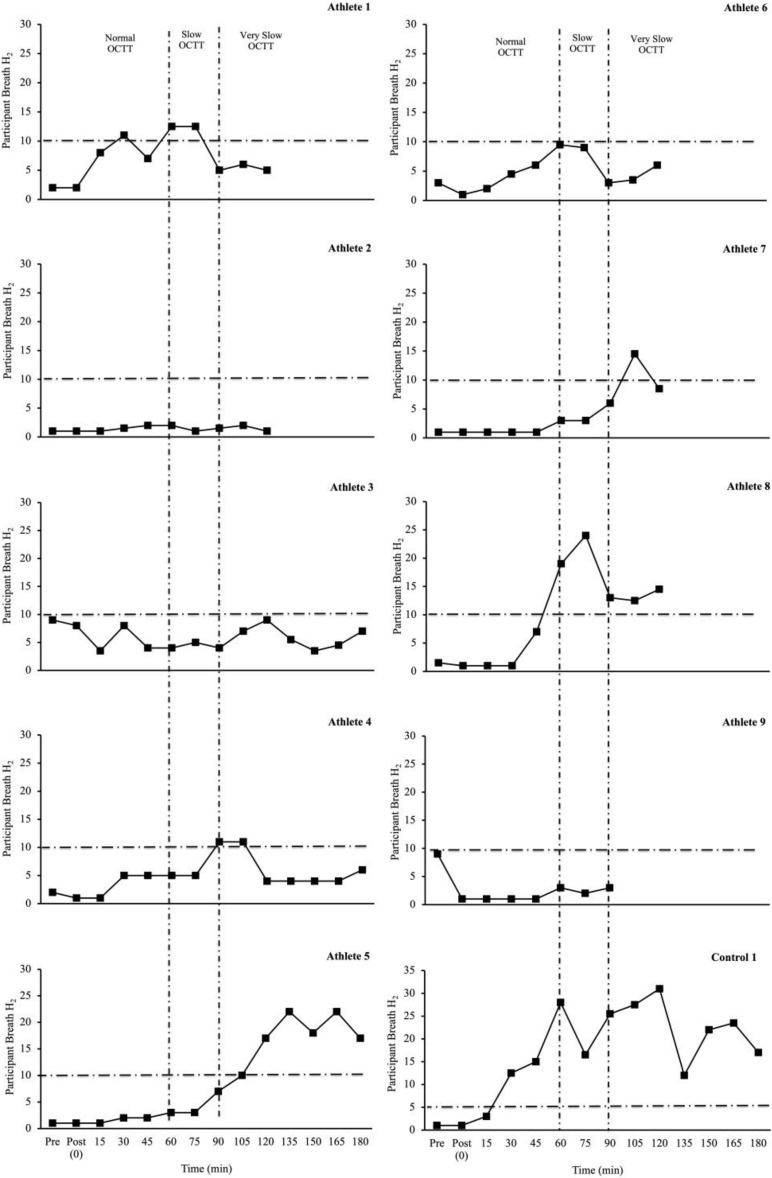
Individual OCTT responses (breath hydrogen turning point (time interval between ingestion of lactulose and rise in breath hydrogen (H_2_) 10 ppm, with two consecutive readings above basal) to laboratory-controlled simulated gastrointestinal assessment during exercise (GastroAxEx) (∼ 2–4 h exertional stress (running ± riding; RPE 13-16) with (∼30–35°C) or without (∼20°C) heat exposure) (*n* = 9). Dash line represents significant rise in breath H_2_ ≥ 10 ppm (Bate et al., 2010). Criteria for suspected exercise-associated gastroparesis and/or paralytic (sub-paralytic) ileus: Normal orocaecal transit response: >10 ppm (vs BL) at 30–60 min post-exercise (1 h post lactulose ingestion). Slow orocaecal transit response: >10 ppm (vs BL) at 60–90 min post-exercise (1 h 30 m post lactulose ingestion). Very slow orocaecal transit response: >10 ppm (vs BL) at 90–120 min post-exercise (2 h post lactulose ingestion). Absent response: No or <10 ppm (vs BL) throughout 2 h recovery period. *N* = 4/9 no turning point i.e., absent response.

Table 2 depicts the physiological and thermoregulatory responses, hydration, blood glucose, and total carbohydrate and fat oxidation rates in response to the GastroAxEx. Table 3 depicts the gastrointestinal integrity and function markers, systemic endotoxin and inflammatory cytokine profiles, GIS during exercise, and feeding tolerance responses to the GastroAxEx. Plasma cortisol increased substantially (Δ > 500 nmol/L) in *n* = 6/7, but not in the control cases. The magnitude of Δ pre- to post-exercise plasma I-FABP concentration was substantial (Δ > 1,000 pg/ml) in *n* = 2/8, but not in the control cases; indicating the exertional (with or without heat) stress exposure resulted in significant epithelial cell (e.g., enterocyte) injury for these athletes, but not in the remaining cases or in the control cases. No substantial change was observed pre- to post-exercise in other markers of gastrointestinal integrity, including plasma claudin, sCD14, LBP, and inflammatory cytokine (IL-1β, TNF-α, IL-6, IL-8, IL-10, and IL-1ra) concentrations. A delayed OCTT response was observed for *n* = 5/9, ranging from 90 to 150 min, and no hydrogen turning point detected in *n* = 4/9. Only one of the control athletes had their OCTT tested and this was classified as a normal response (60 min). Based on OCTT classification criteria, *n* = 2/9, demonstrated a slow OCTT response; *n* = 3/9 very slow OCTT, and *n* = 4/9 an absent response indicative of suspected exercise-associated gastroparesis and/or paralytic (sub-paralytic) ileus (Figure 2). All athletes, except one, experienced GIS during exercise and/or GIS in the post-exercise recovery period. Both of the control cases experienced minimal GIS during and in the post-exercise period. Severe to extreme GIS during exercise was experienced by *n* = 5/9, with *n* = 2/9 having to cease or dramatically reduce their overall work output, in which both required first aid support in the post-exercise period and recovered within the recovery timeframe without any further medical support. Mild to severe nausea was experienced by *n* = 4/9 during exercise, and in *n* = 2/9 during post-exercise recovery. Lack of appetite throughout exercise was reported by *n* = 5/8. Taste fatigue, commonly reported with exercise >4 h (i.e., ultra- endurance), was not reported likely due to the limited duration of exercise. No interest in food was reported by *n* = 4/8. However, tolerance to food (i.e., ability to force feeding) was high in all athletes measured (*n* = 8/8). An interest to drink (i.e., ability to force fluid intake) was reported by *n* = 5/7, with tolerance high in all (*n* = 8/8) athletes.

**TABLE 3:**
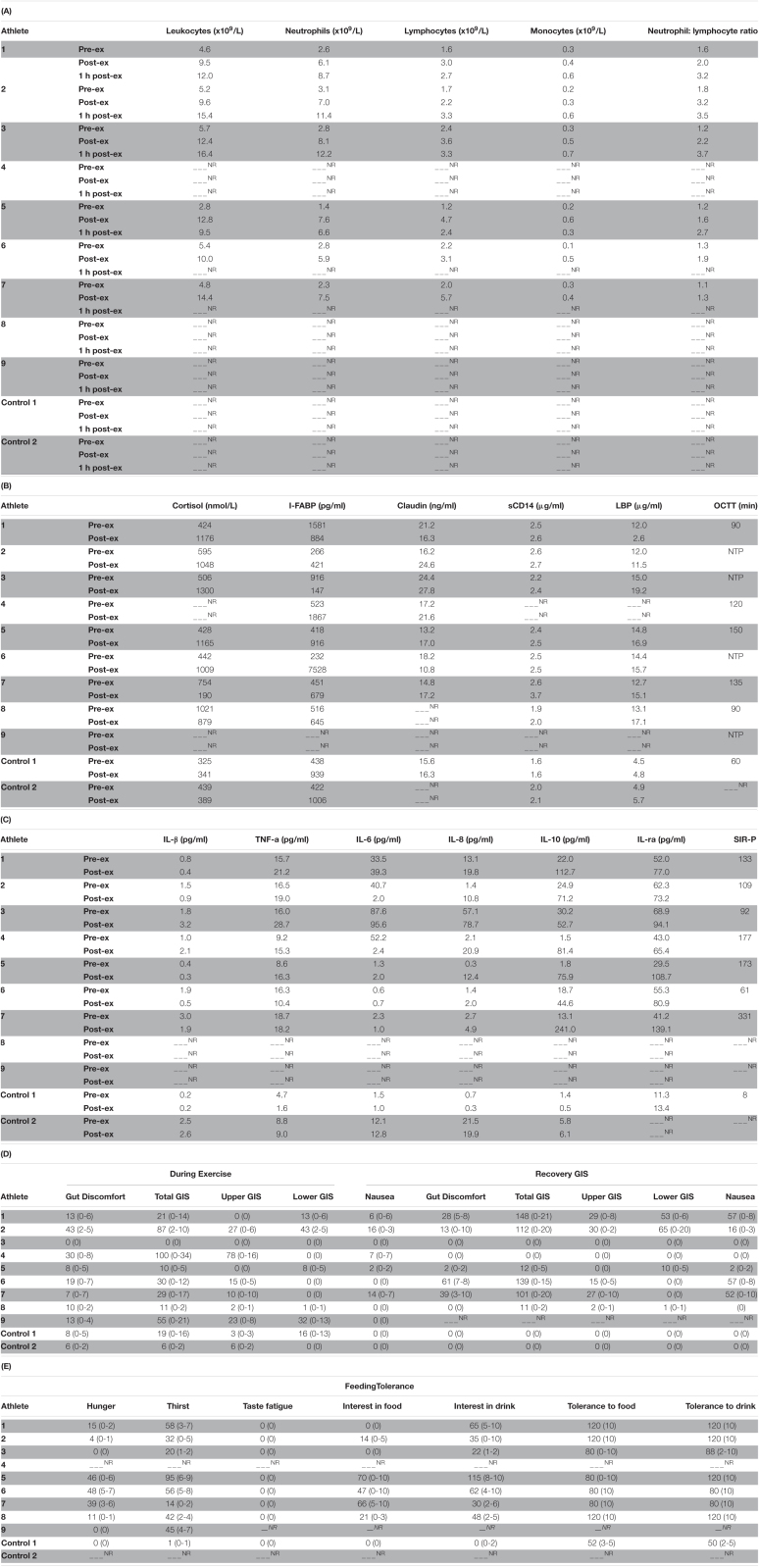
Leukocyte trafficking (**A**), stress hormone response, gastrointestinal integrity and functional responses, systemic endotoxin (**B**), inflammatory cytokine profiles (**C**), gastrointestinal symptoms (GIS) (**D**) and feeding tolerance (**E**) in response to the gastrointestinal assessment during exercise (GastroAxEx) in endurance athletes experiencing EIGS with severe GIS and endurance athletes not experiencing EIGS and presenting minimal GIS (controls).

*Data are presented as individual raw values. For gastrointestinal symptoms (GIS) and feeding tolerance data are presented as overall individual athlete summative accumulation of rating scale point score of measured time periods and individual reported range. NR: not recorded, NTP: no turning point, OCTT: orocecal transit time.*

### EIGS and GIS During Exercise Therapeutic Prevention and Management Plan

Qualitative outcomes are depicted in Table 4. Based on each athlete’s identified proposed causal factors of EIGS and GIS during exercise identified, through the GastroAxEx, an individualised EIGS and GIS during exercise prevention and management intervention protocol was recommended (Table 4). Based on GastroAxEx outcomes and in accordance with previous prevention and management strategies (Costa et al., 2017b, 2019, 2020a; Snipe et al., 2017; Miall et al., 2018; Rauch et al., 2018; Snipe and Costa, 2018a; Bennett et al., 2020; Gaskell et al., 2020, 2021b; Russo et al., 2021a):

• *N* = 9/9, implemented the lead in diet justified based on delayed or absent OCTT.
• *N* = 9/9, gut training and exogenous fuel provision justified due to lack of carbohydrate provisions for required exercise task, or aimed at wanting to improve gut tolerance and/or tolerance to gastric load.
• *N* = 9/9, hydration provision to maintain euhydration during exercise, justified based on total body water losses and/or due to the environmental conditions of the target event/competition.
• *N* = 9/9, thermoregulation, heat acclimation, and cooling (pre- and/or per-, internal and/or external) strategies due to events/competition being conducted in hot/humid conditions and/or they showed poor thermoregulation control/tolerance.
• *N* = 9/9, pre-exercise management to promote bowels being emptied prior to exercise and to allow sufficient time for digestion of nutrients to occur prior to exercise, thus avoiding the gastric-colonic reflux upon food and fluid intake.
• *N* = 7/9, pharmacotherapy (i.e., medical referral for ondansetron prescription) due to significant exercise-associated nausea.
• *N* = 3/9, fat oxidation adaptation training aiming to optimise fat oxidation capacity and reduce reliance on exogenous feeding during exercise.
• *N* = 2/9 pacing strategies aiming to improve management of rapid onset of thermoregulation or physiological strain intolerance.

**TABLE 4:**
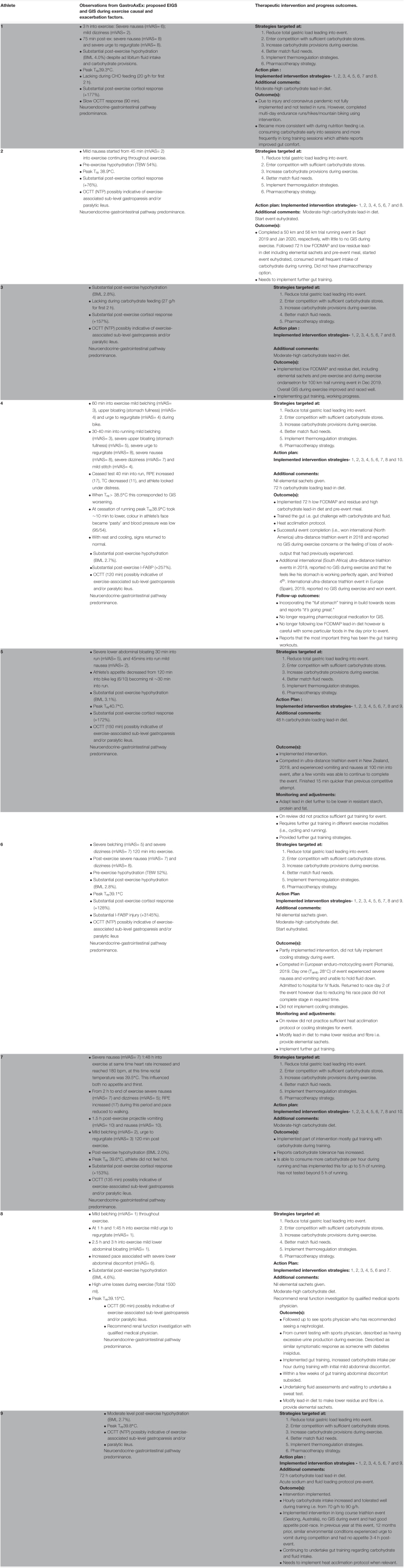
Exercise induced gastrointestinal syndrome (EIGS) and exercise-associated gastrointestinal symptoms prevention and management therapeutic intervention and progress outcomes.

*N* = 8/9, case series athletes reported improvements in their GIS during exercise (Table 4). For example, *n* = 8/9 previously withdrew from competitions due to GIS during exercise; and after intervention. Only *n* = 1/9 reported withdrawal from competition due to similar pre-intervention GIS during exercise. *N* = 7/9 athletes reported completed training and competition with minimal GIS during exercise after intervention. It should be noted, however, that two of these athletes have only been able to implement the intervention in training due to either injury or not entering competition at the time of completion of this case series. *N* = 2 athletes are still needing to implement further intervention strategies and therefore this may be the reason for their more significant ongoing GIS during exercise during competitive events. *N* = 8/9 athletes reported improved feeding tolerance (i.e., able to tolerate higher intakes of carbohydrate and/or fluid feeding during exercise) without substantial gut discomfort.

## Discussion

The current translational research case series aimed to: (1) Clinically assess endurance athletes presenting severe GIS during exercise using retrospective exploration; (2) provide a GastroAxEx using previously established valid and reliable gastrointestinal assessment measurement tools which were used to inform an individualised therapeutic intervention for EIGS and associated GIS; (3) implement individualised therapeutic management interventions; and (4) assess outcomes of therapeutic management plans in training and competition, and adjust accordingly. The current case series of endurance athletes were able to reduce their GIS during exercise in real-world event participation and/or training based on utilising the four-phase approach, in which a tailored GastroAxEx informed by each athlete’s clinical assessment provided sufficient data to develop an individualised therapeutic prevention and management intervention. With the exception of two athletes showing intestinal epithelial injury, no substantial disturbance was observed in the circulatory-gastrointestinal pathway of EIGS in the majority of the case series athletes, including gastrointestinal integrity and systemic endotoxin and immune markers. However, there was disturbance to the neuroendocrine-gastrointestinal pathway of EIGS, indicative of reduced OCTT in all the case athletes. These observations suggest functional issues instigated by EIGS are likely culprits of GIS during exercise in the current cohort, and that targetting interventions to improve these debilitating gastrointestinal functional issues are likely to reduce the incidence and severity of GIS during exercise. Conversely, targetting the circulatory-gastrointestinal pathway specifically with interventions that focus on maintaining the integrity of the intestinal epithelial are unlikely to rectify the GIS during exercise.

### Clinical Assessment of Athletes Presenting With EIGS and Associated GIS During Exercise

Each of the four-phases play a significant role in the prevention and management of EIGS and associated GIS during exercise for the athlete. Phase 1 emphasises the significance of a comprehensive clinical assessment of athletes presenting with EIGS and GIS during exercise in sports dietetic and/or sports medicine practice. This is due to the dynamic and multifactorial nature of EIGS and associated GIS, in which not all athletes present with the same GIS during exercise and/or experience GIS in the same exercise conditions (i.e., exertional stress and environmental conditions). Although the most common reported GIS during competition in the clinical assessment (Phase 1) was nausea and vomiting, negatively influencing feeding tolerance during exercise, two athletes experienced feeding intolerance due to general exercise fatigue accumulation as competition progressed. Some athletes were prone to experience GIS during exercise in the heat, whereas others experienced GIS during exercise in temperate ambient conditions. Within the current case series cohort, onset of GIS during exercise commonly occurred later in exercise (>3 h), appeared rapidly, and before this time athletes reported consuming food and fluid without difficulty. With the rapid onset of GIS experienced at ≥3 h into exercise, the athletes reported an inability to tolerate any food or fluid intake, and in some cases resulting in food and fluid avoidance for the remainder of the competitive event until completion, or even event withdrawal. Endurance and ultra-endurance athletes often report rapid and aggressive onset of GIS during exercise and inability to tolerate feeding when exercise duration is ≥3 h (Pfeiffer et al., 2012; Costa et al., 2016). It is therefore imperative that the sports dietetic or medical practitioner collect comprehensive clinical information of the athlete presenting with EIGS and associated GIS in order to inform the GastroAxEx to best match the scenario resulting in GIS during exercise.

### Gastrointestinal Assessment During Exercise (GastroAxEx): Individual Variation in EIGS and Associated GIS

A substantial amount of both laboratory and field, exploratory or intervention, exercise gastroenterology research has shown large individual variation in EIGS perturbations such as gastrointestinal integrity, function, and systemic markers that can influence GIS during exercise outcomes such as incidence, type, and severity (Gill et al., 2015a, b; Costa et al., 2017b, 2019; Snipe et al., 2017, 2018a,b; Miall et al., 2018; Snipe and Costa, 2018a; Bennett et al., 2020; Gaskell et al., 2020, 2021b; Russo et al., 2021a,b,c). Therefore, it is important that in phase 2 the laboratory controlled GastroAxEx is individualised, as this will subsequently have flow on effects to Phase 3 informing prevention and management strategies of EIGS and associated GIS during exercise. For example, there is individual variability in gastrointestinal function and GIS during diurnal and nocturnal exercise (Gaskell et al., 2021b), and also in feeding tolerance during exercise (Costa et al., 2017b; Miall et al., 2018). This will influence the prevention and management strategy/ies, in which, some athletes may benefit from gut-training during nocturnal exercise, while others may benefit more from gut-training strategies during diurnal exercise. Other athletes may have varying feeding tolerance levels that require an individualised gut challenge protocol.

In the current athlete case series the following outcomes were observed: (1) The athlete that presented the greatest intestinal epithelial injury (i.e., Δ plasma I-FABP concentration: 7,296 pg/ml, Athlete-6) was found to have an absent OCTT response indicative of suspected exercise-associated gastroparesis and/or paralytic (sub-paralytic) ileus. Proposed Δ pre- to post-exercise plasma I-FABP concentration reflecting magnitude of clinical relevance (i.e., comparative values observed in gastrointestinal inflammatory or functional disease/disorders and/or symptomatic characteristics warranting medical management) consistently associated with perturbed systemic endotoxin and inflammatory profiles and GIS appears to be ≥1,000 pg/ml (Costa et al., 2017b, 2020b). The athlete also experienced severe belching and dizziness during exercise and severe dizziness and nausea post-exercise. The magnitude of injury observed in this athlete after running for 2 h at RPE of 15 in 33°C, even with carbohydrate feeding in the first 1 h, exceeds that of previous exertional- stress research (2 h running at 60–70% V̇O_2__max_) with (T_amb_ 30–35°C) and without (∼20°C) heat exposure (plasma I-FABP concentration mean range 82–2,269 pg/ml) (Snipe et al., 2017, Snipe et al., 2018a,b; Costa et al., 2019; Gaskell et al., 2020, 2021b), and 3 h running protocol with greater carbohydrate intake (90 g/h 2:1 glucose:fructose, 10% w/v) in first 2 h (I-FABP ≤ 1,000 pg/ml) (Costa et al., 2017a). (2) The athlete that presented the greatest perturbations to the endotoxin (i.e., Δ plasma sCD14 and LBP concentration: 1.1 μg/ml and 2.4 μg/ml, respectively, Athlete-7) and inflammatory cytokine profile (i.e., SIR-P: 331 arb.unit attributed to a pronounced anti-inflammatory response (i.e., + 1749% IL-10 and + 238% IL-1ra), with adjunct leucocytosis (combined neutrophilia and lymphocytosis), Athlete-7) was found to have a very slow OCTT, experienced severe dizziness and nausea during exercise that influenced workload needing to be reduced, and also reported low interest and tolerance to food and fluid during exercise. After exercise this athlete experienced projectile vomiting, nausea, belching and urge to regurgitate. The magnitude of endotoxin perturbation and inflammatory cytokine profile response observed in this athlete after running for 2 h at RPE 16 in T_amb_ 32°C is significantly greater compared to previous laboratory exertional-heat stress research (2 h running at 60% V̇O_2__max_ in T_amb_ 36°C) (Δ plasma sCD14 0.38 μg/ml and LBP < 0.01 μg/ml) (Gaskell et al., 2020) and (2 h running at 60% V̇O_2__max_ in T_amb_ 30–35°C) (SIR-P mean range 116–245 arb.unit) (Snipe et al., 2018a,b). Furthermore, this athlete’s pronounced anti-inflammatory response (Δ plasma IL-10 228 pg/ml and IL-1ra 98 pg/ml) exceeds that observed in ultra-marathon field studies, such as a five stage 230 km event (overall mean IL-10 10 pg/ml and IL-1ra 112 pg/ml) and a 24 h continuous ultra-marathon (overall mean IL-10 11 pg/ml) (Gill et al., 2015a, b), in which a pro-inflammatory cytokinaemia was observed. (3) The athlete that presented the greatest GIS during exercise (Athlete-4) was found to have a slow OCTT, experienced mild belching, upper bloating and urge to regurgitate during cycle exercise; then mild belching and stitch, plus severe upper bloating, urge to regurgitate and nausea during running exercise, and subsequently had to cease exercise due to extremely severe upper GIS during exercise. The two control cases that presented minimal GIS during and in the post-exercise period also presented with minimal disturbance to gastrointestinal integrity, function (where measured), systemic endotoxaemia and inflammatory profile response; but magnitude of responses were of no clinical consequence and not sufficient to impede exercise workload and warrant exercise cessation or withdrawal. In comparison, previous laboratory exertional stress models, all study participants have completed the exercise protocol (Costa et al., 2017a, 2019; Snipe et al., 2017, 2018a,b; Miall et al., 2018; Snipe and Costa, 2018a; McCubbin et al., 2019; Gaskell et al., 2020, 2021b; Russo et al., 2021b) i.e., no GIS-associated withdrawal, except in Gaskell et al. (2021b), in which *n* = 3 participants did not complete the nocturnal exercise protocol (3 h running at 60% V̇O_2__max_ in T_amb_ 23°C) due to severe GIS symptoms including nausea, projectile vomiting and explosive bowel movements. (4) Athletes with absent turning point in OCTT experience either no appetite during exercise (*n* = 3/4) or a moderate level of appetite during exercise (*n* = 1/4), *n* = 2/4 did not experience any significant GIS during exercise or post-exercise, and *n* = 2/4 experienced either mild or severe GIS during exercise such as either belching, dizziness or nausea. All participants in this case series experienced a delayed OCTT response, this is in contrast to Gaskell et al. (2021b) in which 39 and 62% of participants presented with delayed OCTT in response to diurnal and nocturnal exercise respectively. The exertional stress model differed between studies with the case series involving 2–4 h of running or a combination of cycling and running in the daytime in warm to hot conditions (*n* = 6/9) or thermoneutral conditions (*n* = 3/9) with RPE 13–17. In contrast, the exercise stress model in Gaskell et al. (2021b) involved diurnal and nocturnal running for 3 h at 60% V̇O_2__max_ in temperate conditions with RPE 12. Due to large individual variation in factors exacerbating GIS during exercise in athletes there is no one standard approach and therapeutic intervention. Practitioners should be warned against any one particular prevention and management strategy claiming to resolve EIGS and/or GIS during exercise, as each individual case is different and unique to the individual. There are many examples of claims being made in relation to nutrition supplementation for the management of EIGS and/or GIS during exercise, such as the use of probiotics for gut health in athletes, however, research evidence efficacy does not support its role as a therapeutic intervention in EIGS and GIS during exercise (Möller et al., 2019). Other examples of nutrition supplementation lacking evidence in this area are amino acids (e.g., glutamine, L-arginine, L-citrulline, glyceine, and tyrosine), bovine colostrum, anti-oxidants, curcumin, and nitrate (Costa et al., 2017b, 2020b).

When designing the GastroAxEx, it may not always be possible to identically replicate the exercise (i.e., modality or exercise intensity and duration). However, the most important point observed is to simulate the exertional stress, getting it as close as possible to the affected individual’s experience, and ensuring it is sufficient to induce EIGS and prompt GIS during exercise. For example, one of the case series athletes experienced EIGS during enduro-motorcycling events, suggested to be due to dehydration and heat stress. The athlete also participated in marathon running events. Therefore, a running exertional model was used to induce similar dehydration and thermoregulatory strain due to the logistics of simulating an enduro-motorcycling bout in laboratory-controlled conditions. When conducting the GastroAxEx, it is important to mimic the real-life scenario as close as possible leading up to EIGS and GIS during exercise (i.e., athletes should follow their typical lead-in race/event diet, pre-exercise meal and during exercise nutrition). This will then help identify the main causal and exacerbation factors of EIGS, and subsequent GIS during exercise, which will inform the individualised therapeutic intervention for the prevention and management of EIGS and GIS during exercise.

### Individualised Therapeutic Intervention Targetted at EIGS and GIS During Exercise

Phase 3 involves an individualised therapeutic intervention targetted at EIGS and GIS during exercise, that informed by the GastroAxEx. Once the causal and exacerbator factors are identified the intervention can be determined. Without an understanding of the causal pathway the prevention and management strategies will be non-specific and experimental. For example, the professional triathlete trialled a number of different strategies in order to manage his EIGS and associated GIS during exercise, such as a low FODMAP diet, low carbohydrate and high fat diet and changing the type of race nutrition in training and competition without success. These strategies were not based on any objective data but rather anecdotal and testimonial. As can be seen in Table 4, the causal pathway and exacerbator factors of EIGS and GIS during exercise for him did not relate to these specific strategies. An interesting and important observation in the current case series was that the neuroendocrine-gastrointestinal pathway of EIGS appeared to be the predominant causal factor of GIS during exercise in the entire athlete cohort. All athletes were identified to have a delayed orocecal transit, suggesting that gastrointestinal function had become impaired. Implementing prevention and management strategies targeted at the circulatory-gastrointestinal pathway such as promoting maintenance of splanchnic and villi microvascular perfusion via small and regular carbohydrate feeding in isolation would likely not resolve EIGS and GIS during exercise. In consideration of this GastroAxEx outcome, a common therapeutic strategy used in all athletes was a 48 h dietary control comprising of a low FODMAP, fibre and residue intake with the aim to reduce gastrointestinal burden and reduce the risk of malabsorbed nutrients arriving at the ileum potentially supressing gastrointestinal motility through braking mechanisms and consequently exacerbating GIS during exercise (Layer et al., 1990; van Citters and Lin, 2006; Shin et al., 2013; van Avesaat et al., 2015; Costa et al., 2017a; Miall et al., 2018; Gaskell et al., 2020). Other shared themes of the therapeutic management plan amongst athletes was gut-training, as previously described by Costa et al. (2017a) (i.e., repetitive gut-challenge), advised based on identifying that each athlete had impaired gastrointestinal function. Gut-training has been shown to attenuate EIGS and GIS during exercise (neuroendocrine-gastrointestinal pathway), resulting in improved exercise performance (i.e., 1 h distance test) (Costa et al., 2017a; Miall et al., 2018). Small and frequent feeding of a carbohydrate solution was recommended to all athletes based on this method of carbohydrate ingestion and delivery helping maintain a constant and steady intragastric pressure, facilitating an enhanced gastric emptying rate and better stomach comfort (Noakes et al., 1991; Lambert et al., 2008). Fluid tolerance training involving repeated exposure to ingesting fluid has been shown to significantly improve gastrointestinal comfort, possibly related to increased gastric tolerance (Goulet, 2011). Therefore, fluid tolerance training was advised in athletes identified as having significant body water losses (i.e., dehydration) during exercise despite water provisions aimed at maintaining euhydration (i.e., body water losses through sweating > water provisions and bioavailability). Athletes presenting with significant nausea were recommended pharmacotherapy intervention such as oral use of the antiemetic drug ondansetron as it has been used successfully for alleviating this symptom in some competitive situations and is commonly found at endurance races for treating nausea and vomiting (Hoffman et al., 2014). Therapeutic intervention for the prevention and management of EIGS and GIS during exercise is dependent on the causal and exacerbation factor(s) of EIGS that are identified through using appropriate (i.e., validated and reliability checked) assessment techniques (Gaskell et al., 2021a).

### Monitoring and Readjustment of Intervention

Phase 4 involves monitoring and adjusting the intervention based on the athlete’s outcomes and feedback in training and competition. The intervention is not static and will evolve over time based on each individual’s response and dependent on environmental conditions. The success of the intervention is largely influenced by this phase. Based on the qualitative data reported by each athlete, the therapeutic intervention appeared to help improve management of their GIS during exercise. It was recognised, however, that some athletes did not completely understand the importance of each strategy and the contribution each strategy played within the template intervention, and in some cases athletes did not fully implement the proposed strategy/ies (Table 4). For example, the enduro-motorcyclist, who raced in an international enduro-cycle event based in Romania, did not undertake sufficient heat acclimation prior to the event, and did not implement the complete cooling strategy recommended. Other athletes did not allow sufficient time for gut-training or did not undertake sufficient protocols (i.e., intake volume, nutrient density, and/or texture) to challenge the gut, prior to the target competition. Without monitoring the athletes this information would not have been known and therefore their ongoing struggle with EIGS and GIS during exercise would continue. The current case series highlights the importance of the collaborative relationship between the practitioner and athlete in which two-way communication and feedback is imperative to the success of the intervention. The athlete needs to invest equally in each phase and be willing to practice and adapt the therapeutic intervention as they learn their response and due to the dynamic nature of EIGS and exacerbation factors. The practitioner also needs to invest equal time and attention to each phase and understand that they will need to adjust the therapeutic intervention based on the athlete’s feedback and response, additional GastroAxEx may be warranted.

### Cohort Outcomes of Therapeutic Intervention of EIGS

Significant and substantial positive outcomes were reported by the majority of the case series cohort (Table 4). For example, the professional long course triathlete implemented the therapeutic intervention in the lead-up to and during a key ultra-distance triathlon event, and for the first time in multiple ultra-distance triathlon event attempts, spanning several years, resulting in many event withdrawals, did not experience their typical GIS during exercise that would normally dramatically impair their work output. The triathlete won the ultra-distance triathlon event, a significant experience for the triathlete as numerous dietary interventions to help manage their GIS during exercise had been trialled without positive outcomes, and in some cases negative outcomes. These included increasing carbohydrate and fluid ingestion in training without objective guidance, manipulating the amount and composition of carbohydrate and fluid during competition to within tolerance levels that including training and racing with a carbohydrate hydrogel product (McCubbin et al., 2019), and a 32-weeks low carbohydrate high fat (LCHF) diet (Mujika, 2019). In the instance of using the hydrogel product, GIS during exercise issues were still present; and following the LCHF diet a negative outcome was reported (Mujika, 2019). Another example is of an age-group long course triathlete who previously experienced no tolerance to feeding and drinking, nausea and projectile vomiting during the run leg of triathlon events with loss of appetite post-event. Since gastrointestinal intervention practice, the athlete was able to compete and complete in ultra-distance triathlon event in a hot and humid environment (Kona, Hawaii, United States) without any significant GIS during exercise. The second ultra-distance triathlon event (Geelong, Victoria, Australia), the athlete competed, since implementation of the therapeutic intervention, resulted in an event completion without any significant GIS during exercise and tolerated food and fluid well throughout the race and immediately post-race. Twelve months prior, in this same race, and similar environmental conditions, the athlete experienced urge to vomit and no appetite for 3–4 h post-race. It is important to also highlight that limited positive outcomes were seen in athletes that did not fully and consistently follow the therapeutic intervention.

## Conclusion

Considering the dynamic and multifactorial nature of EIGS and associated GIS during exercise, an individualised therapeutic approach is warranted in which comprehensive clinical assessment is gathered, an individual tailored laboratory controlled GastroAxEx is designed and conducted informing the therapeutic prevention and management intervention which can be adjusted based on monitoring the individual athlete’s response to the intervention. This translational research practice resulted in decreased GIS during exercise incidence and severity during training and competition, and resulted in substantially improved work output, reduced exercise cessation and/or withdrawal in most cases.

## Data Availability Statement

The raw data supporting the conclusions of this article will be made available by the authors, without undue reservation.

## Ethics Statement

The studies involving human participants were reviewed and approved by Monash University Human Research Ethics Committee. The patients/participants provided their written informed consent to participate in this study.

## Author Contributions

RC was the chief investigator and lead practitioner of this translational research. RC and SG contributed toward the original research idea and development of the experimental design, contributed to the draft preparation of the manuscript. RC, SG, and CR contributed to the various aspects of data collection, sample collection, and analysis, contributed to the processing and analysis of the raw data. All authors reviewed and approved the final manuscript.

## Conflict of Interest

RC is the lead Sports Dietetic and Extremes Physiology practitioner for the clinic. CR is the principal Sports Dietetic practitioner for the clinic service offerings. The remaining author declares that the research was conducted in the absence of any commercial or financial relationships that could be construed as a potential conflict of interest.

## Publisher’s Note

All claims expressed in this article are solely those of the authors and do not necessarily represent those of their affiliated organizations, or those of the publisher, the editors and the reviewers. Any product that may be evaluated in this article, or claim that may be made by its manufacturer, is not guaranteed or endorsed by the publisher.
